# Xenotransplantation: a consistent perspective

**DOI:** 10.1590/0100-6991e-2022EDIT01

**Published:** 2022-02-18

**Authors:** SILVANO MÁRIO ATTÍLIO RAIA

**Affiliations:** 1 - Universidade de São Paulo - São Paulo - SP - Brasil

**Keywords:** Transplants, CRISPR-Associated Protein 9, Ethical Theory, Transplante Heterólogo, Proteína 9 Associada à CRISPR, Ética

## Abstract

Are presented results of experimental pig kidney xenotransplantation in Brazil, which aims to reduce the waiting list mortality due to shortage of organs. Recent clinical results obtained abroad are commented.

## RESUMO

Apresentam-se resultados de xenotransplante suíno de rim experimental no Brasil que visa reduzir as listas de espera nas quais falecem muitos inscritos à espera do transplante. Comentam-se os recentes resultados clínicos obtidos no exterior.

In all transplant centers there is a lack of organs cause of the waiting lists. Many patients die before transplanted. Then, an attempt has been made to develop methods to obtain additional organs. The most promising is xenotransplantation, which proposes to transplant animal organs into humans.

Pigs (*Sus scrofa domesticus*) are the most suitable donors. Easy to handle, omnivorous, physiology similar to ours, organs of compatible size, short gestation period and numerous litters.

However, their use as organ donors depends on changes in their genome, such as inactivation (knock out) of the genes responsible for some sugars production, which causes hyperacute rejection and the addition (knock in) of human genes that produce substances capable of modulate chronic rejection.

In 2014, the CRISPR-Cas9 technique facilitated genetic edition. It easily allows to block, add or subtract genes from any living organism.

With this technique, encouraging experimental results of xenotransplantation have been reported. In 2016, Mohammad Mohiuddin et al. (Nature) reported pig heart xenotransplantation in baboons, with a survival of up to 2.5 years. In 2017, Hayato Iwase et al. (Xenotransplantation) reported, also in baboons, pig kidney xenotransplantation with survival of up to 8 months. The animals were sacrificed for other causes (lack of access) with excellent renal function (sic).

The risk of transmission of PERVs (pig endogenous retro virus), which was the reason for the ban on pig xenotransplantation, codified by the Changsa declarations of 1998 and 2006, still needed to be resolved.

However, in 2017, Church et al., from Harvard University, published in Science the inactivation of the 62 loci of PERVs, producing virus free pigs, opening perspectives for the clinical use of the new method.

Considering all these facts and the demand in Brazil, where, in 2019, 133,000 patients were on hemodialysis, of which 59,850 were eligible for kidney transplantation, but only 6,000 were transplanted (9.8%) and that 22,000 died while waiting the procedure (1 death every 6 hours), it seemed justified to try to develop the new method among us as well.

Thus, together with a multidisciplinary team from USP, which includes professors Jorge Kalil, Elias David Neto and Maria Rita Passos Bueno, we are developing a project to systematize pig kidney xenotransplantation.

The agreement of ethical, religious and legal authorities for its clinical use in Brazil was obtained. In this sense, it is worth noting that, in 2021, a group from the Faculty of Medicine of São José do Rio Preto led by Dr Cinthia Pavan showed, in Brazil, an acceptance of pig xenotransplantation above 80% among 50 patients enrolled on the waiting list and 50 liver transplants.

At the Center for Human Genome and Stem Cell Studies (CEGH-CEL) at USP, we have already completed the programmed gene editing phase: the 3 genes that produce immunogenic sugars - CMAH, GGTA1, β4GalNT2 - were inactivated in fibroblasts (knock out) which were transferred to unmodified nucleus of embryos in the zygote phases. At the same time, CD55 and CD47 genes were added (knock in). Then, the embryo thus modified will be implanted laparoscopically in the fallopian tube of wild sows. These will produce litters of genetically modified pigs whose organs can be transplanted into humans.

Now it is necessary to build a pig facility, complying with the strict national and international regulation of biosafety (NB2) to breed pigs whose organs can be transplanted into humans.

Considering that recently data report also good results with heart, skin and cornea xenotransplantation, we have programmed the following pre-clinical experiments during the 18 months planned for the construction of the pig facility.

The genetically modified pigs will be bred, until they reach 40kg, in a normal hygienic animal facility specifically built in the Faculty of Medicine of the University of São Paulo.

Their kidneys and heart will be tested for 18 hours in isolated perfusion systems in alternating experiments with pig blood and human blood. The results will be evaluated by biochemical measurements in successive samples collected in the perfusate and by anatomopathological study of serial biopsies and study of the organs as a whole after the end of the perfusion.

Grafts obtained with a dermatome and stored in the HC Skin Bank in saline at 25ºC for progressively longer periods will be used for skin grafts to systematize their use in the treatment of severe burns. The grafting will be done alternatively in wild animals and in genetically modified animals.

The cornea will be removed after ocular enucleation of the donor pig, then preserved in Optisol GS at 4ºC for up to 14 days and finally implanted alternatively in wild pigs and in genetically modified pigs.

Depending on the results of the isolated perfusion of the kidney, genetically modified in our laboratory, with human blood, one or two kidney xenotransplantations will be performed in human recipients with brain death (similar to what was recently carried out in New York).

Finally, the development of this project made it clear that it is not just an academic-scientific project, but an attempt to modify several concepts, including a medical-social one, that is, the production and use of animal organs for transplantation in humans. This project required solving unexpected difficulties, such as, among others, obtaining authorization to breed pigs within the urban perimeter, authorization to dispose of genetically modified animal waste in the municipal sewer, obtain newly extracted pig ovaries at slaughterhouses close to the Genome Center and obtain 1500 female pigs of 100 kg per year.

The pig heart xenotransplantation performed in Maryland (USA) on January 17, 2022, opens perspectives for the clinical start of kidney, skin and cornea xenotransplantation as well. In fact, the favorable evolution of the patient until the ninth day (date of writing of this article) shows that the hyperacute rejection, the cause of death of the xenotransplanted child in 1984, was overcome.

Overcoming all the obstacle of this project has been a great challenge. However, experience shows that in innovative projects, each completed stage allows a greater angle of vision that can both identify unforeseen difficulties, which delay the achievement of the final objective, or show shortcuts that allow it to be anticipated. For the benefit of many, let’s hope that for our project, the second alternative will occur.

